# Distribution of genetic alterations in high-risk early-stage cervical cancer patients treated with postoperative radiation therapy

**DOI:** 10.1038/s41598-021-90139-0

**Published:** 2021-05-19

**Authors:** Naoya Murakami, Yuka Asami, Hiroshi Yoshida, Daisuke Takayanagi, Sou Hirose, Ikumi Kuno, Kazuaki Takahashi, Maiko Matsuda, Yoko Shimada, Shotaro Yamano, Kuniko Sunami, Takayuki Honda, Tomomi Nakahara, Tomoko Watanabe, Kae Okuma, Takafumi Kuroda, Takashi Kohno, Tomoyasu Kato, Kouya Shiraishi, Jun Itami

**Affiliations:** 1 Department of Radiation Oncology, National Cancer Center Hospital, 5-1-1 Tsukiji, Chuo-ku, Tokyo, 104-0045 Japan; 2 Division of Genome Biology, National Cancer Center Research Institute, 5-1-1 Tsukiji, Chuo-ku, Tokyo, 104-0045 Japan; 3 Department of Obstetrics and Gynecology, Showa University School of Medicine, Tokyo, Japan; 4 Department of Diagnostic Pathology, National Cancer Center Hospital, Tokyo, Japan; 5 Department of Obstetrics and Gynecology, The Jikei University School of Medicine, Tokyo, Japan; 6 Department of Medical Oncology, Osaka International Cancer Institute, Osaka, Japan; 7 Japan Bioassay Research Center, Japan Organization of Occupational Health and Safety, Kanagawa, Japan; 8 Department of Respiratory Medicine, Tokyo Medical and Dental University, Tokyo, Japan; 9 Division of Carcinogenesis and Cancer Prevention, Department of Immune Medicine, National Cancer Center Research Institute, Tokyo, Japan; 10 Department of Gynecologic Oncology, National Cancer Center Hospital, Tokyo, Japan

**Keywords:** Genomics, Radiotherapy, Cervical cancer, Oncogenes, Cancer, Tumour heterogeneity

## Abstract

Somatic genetic alteration analysis was performed for post-hysterectomy high-risk early-stage uterine cervical cancer patients who underwent post-operative radiation therapy. Post-operative radiation therapy was performed for patients with pathological features of pelvic lymph node metastasis, parametrium invasion, or positive vaginal margin, which corresponded to the post-operative high-risk category. DNA was extracted from paraffin-embedded surgical specimens, and 50 somatic hotspot genetic alternations were detected using Ion AmpliSeq Cancer Hotspot Panel. The existence of actionable mutation was assessed based on OncoKB evidence level > 3A. Between January 2008 and November 2019, 89 patients who underwent abdominal radical hysterectomy followed by post-operative radiation therapy were identified. The follow-up period for living patients was 82.3 months (range 9.3–153.9), and the 5-year relapse-free survival and overall survival rates were 72.6% and 85.9%, respectively. The most frequently detected somatic mutation was *PIK3CA* (26 [29.2%] patients); however, no prognostic somatic genetic alterations were identified. Actionable mutations were detected in 30 (33.7%) patients. Actionable mutations were detected in approximately one-third of patients, suggesting that precision medicine can be offered to patients with post-operative high-risk uterine cervical cancer in the near future.

## Introduction

Since the development of an effective screening system and prophylactic vaccines against oncogenic human papillomaviruses (HPVs), which are the primary cause of uterine cervical cancer^1,2^, the incidence of uterine cervical cancer has drastically decreased in most developed western countries^3^. However, uterine cervical cancer is a big public health problem in Japan and in many developing countries^4^; annually, 20,000 new cases and approximately 2600–2900 deaths are reported^5,6^, and the rate of screening and vaccination for HPV is low in Japan.

Among early-stage post-hysterectomy uterine cervical cancer patients, post-operative pelvic radiation therapy improves pelvic control in patients with intermediate-risk prognostic factors, while concurrent chemoradiation therapy (CCRT) improves overall survival in patients with high-risk prognostic factors^7,8^.

A comprehensive genomic investigation of uterine cervical cancer has revealed several novel somatic genetic alterations that are potential therapeutic targets for cervical cancer patients^9^. However, thus far, only few studies have performed somatic genetic alteration analysis in high-risk early-stage cervical cancer Asian females. The purpose of this study was to investigate the distribution of somatic genetic alterations and the frequency of actionable mutations in Japanese patients with post-hysterectomy high-risk early-stage uterine cervical cancer who underwent post-operative radiation therapy.

## Methods

Pre-operative staging work-up for uterine cervical cancer included physical examination; chest, upper abdominal, and pelvic computed tomography (CT) with contrast enhancement, and pelvic magnetic resonance imaging (MRI). All patients were re-staged according to the International Federation of Gynecology and Obstetrics (FIGO) 2018 staging system for this analysis. According to the Japan Society of Gynecologic Oncology guidelines 2017 for the treatment of uterine cervical cancer^10^, patients aged < 65 with FIGO stage IB2–IIB disease are candidates for abdominal radical hysterectomy. The treatment strategy for each patient was discussed in our Gynecological Tumor Board, comprising gynecologic oncologists, radiation oncologists, radiologists, pathologists, and medical oncologists. Abdominal radical hysterectomy was performed according to a previously reported method^11^. In brief, the anterior, medial, and posterior retinaculum uteri were isolated and divided. After dividing all three retinacula uteri, paravaginal tissue and the vaginal wall were incised, and the uterus and adnexa were removed en masse. Dissection of regional lymph nodes (LNs; common iliac LNs, external iliac LNs, internal iliac LNs, obturator LNs, sacral LNs, and cardinal ligament LNs) was also performed.

The indication criterion for post-operative pelvic radiation therapy in our institution is the presence of a high-risk feature, such as positive margin, pelvic lymph node metastasis, or parametrium invasion^12^. Before 2010, pelvic radiation therapy was delivered via three-dimensional conformal radiation therapy (3D-CRT) with a 4-field box technique. Because the incidence of ileus was as high as 27% in our institution after abdominal radical hysterectomy and adjuvant pelvic radiation therapy alone with 3D-CRT, concurrent chemotherapy was not administered because it could potentially increase the occurrence of ileus even higher despite a phase 3 clinical trial showing overall survival benefits adding concurrent chemotherapy^8^. Since 2010, intensity-modulated radiation therapy (IMRT) was used to reduce bowel toxicity; after the introduction of IMRT, the bowel obstruction rate was decreased to 7.5%^12^. Then, it was considered that it would be safe to add concurrent chemotherapy with IMRT, and since 2015, concurrent weekly cisplatin (CDDP) (40 mg/m^2^) has been used for the treatment of post-operative high-risk early-stage uterine cervical cancer patients.

The pelvic radiation dose was 50 Gy in 25 fractions. The typical radiation field included the external, common, and internal iliac LNs, presacral LN, obturator LN, vaginal cuff, and paracolpium. If a pathologically positive para-aortic LN was present, extended-field portals up to the Th12/L1 interspace was applied. Patients with only positive vaginal margin received vaginal intracavitary brachytherapy alone without pelvic external beam radiation therapy. For patients with both LN metastasis/parametrium invasion and positive vaginal margin, vaginal intracavitary brachytherapy was delivered after whole pelvic radiation therapy.

According to previous reports, *PIK3CA* mutations are detected in 30% cervical cancer patients. Assuming a recurrence in 30% of 100 cervical cancer cases, the power of an odds ratio of 3 was ≥ 0.80. Therefore, 107 patients were enrolled in this study. Surgically removed paraffin-embedded specimens were used for somatic genetic alteration analysis. Genomic DNA was extracted from formalin-fixed, paraffin-embedded (FFPE) tumor tissues using the QIAamp DNA FFPE tissue kit (Qiagen, Hilden, Germany), according to the manufacturer’s instructions. Purified genomic DNA obtained from tumor tissues (50 ng) was used for library construction using the Ion AmpliSeq Cancer Hotspot Panel v2 (Thermo Fisher Scientific, Waltham, MA, USA) that targets approximately 2800 COSMIC mutational hotspot regions of 50 cancer-related genes. Sequencing was performed on the Ion Proton platform (Thermo Fisher Scientific). Samples with a mean read depth of coverage of > 1000 and a base quality score of 20 (i.e., with a ≤ 1% probability of being incorrect), which accounted for 90% of the total reads, were selected for sample quality control. Data analysis was performed using the Torrent Suite Software v5.0.4 (Thermo Fisher Scientific). First, somatic mutations were selected using the following criteria: (1) the variant allele frequency of somatic mutations was > 4% in tumor tissues; (2) single nucleotide polymorphisms were removed if they showed a threshold allele frequency value of ≥ 0.01 in either the NHLBI GO Exome Sequencing Project (ESP6500; http://evs.gs.washington.edu/EVS/) or the Integrative Japanese Genome Variation Database (iJGVD, 20151218; https://ijgvd.megabank.tohoku.ac.jp/)^13^; (3) mutations were registered as “pathogenic/likely pathogenic variants” in ClinVar^14^ or “oncogenic/likely oncogenic variants” in the TCGA dataset^9,15^ (retrieved data from cBioPortal; http://www.cbioportal.org/). Then, all selected variants were judged by manual inspection using the Integrative Genomics Viewer (http://www.broadinstitute.org/igv/)^16^.

Copy number alterations were detected with real-time genomic polymerase chain reaction (PCR) using the TaqMan copy number assay and the ABI 7900HT real-time PCR system (Applied Biosystems). Among 50 targeted genes, four genes, i.e., *PIK3CA*, *ERBB2*, *PTEN*, and *STK11*, were selected in the Ion AmpliSeq Cancer Hotspot Panel v2; the frequency of copy number alterations in these four genes was > 5% in the TCGA dataset of cervical cancer patients within cBioPortal. All TaqMan probes, including *PIK3CA* (ID Hs02202946_cn), *ERBB2* (ID Hs02803918_cn), *PTEN* (ID Hs05128032_cn), *STK11* (ID Hs04013006_cn), and *RNaseP* (cat. no. 4403328; reference), were purchased from Thermo Fisher Scientific. Genome data were analyzed using the ABI PRISM 7900HT Sequence Detection Software CopyCaller v2.1 (Thermo Fisher Scientific) for copy number analysis. Copy number amplification was defined as the process resulting in the presence of > 8 copies, while copy number loss was defined as the process resulting in the presence of < 1.2 copies, as previously reported^17^.

HPV genotyping was performed using Sanger sequencing. Genomic DNA (10 ng) was amplified via PCR using Taq DNA polymerase (Takara Bio Inc., Shiga, Japan); two pairs of consensus primers (HPVpU-1M: TGTCAAAAACCGTTGTGTCC and HPVpU-2R: GAGCTGTCGCTTAATTGCTC); PCR Human Papillomavirus Typing Set (Takara Bio Inc.)^18^, i.e., at the E6 and E7 homologous regions of HPV (228–268 bp); and other general primers (HPV-GP5: TTTGTTACTGTGGTAGATAC and HPV-GP6: GAAAAATAAACTGTAAATCA)^19^. PCR products were purified using NucleoSpin Gel (Takara Bio Inc.) or the PCR Clean-up (Takara Bio Inc.) kit. Sanger sequencing was performed using the ABI 3130xl DNA Sequencer (Applied Biosystems), in accordance with the manufacturer’s instructions. The similarity between the obtained sequences and various HPV genotypes in the GenBank database was determined using BLAST (https://blast.ncbi.nlm.nih.gov/Blast.cgi) analysis.

To clarify the frequency of HPV-positive results in the samples, we performed in situ hybridization assay for HPV detection using HPV-III High-Risk probes (Roche Diagnostics, Mannheim, Germany) in accordance with the manufacturer’s instructions. This assay can detect high-risk HPV genotypes, including HPV-16, HPV-18, HPV-31, HPV-33, HPV-35, HPV-45, HPV-52, HPV-56, HPV-58, and HPV-66, in cervical cancer specimens.

OncoKB^20^ (https://oncokb.org/#/; oncokb-annotator; commit 8910b65 on June 29, 2019) is a precision oncology knowledge database that contains information about the effects and treatment implications of specific genomic alterations in cancer patients. Somatic mutations and copy number alterations were categorized into four evidence levels. In the present study, genetic aberrations with evidence levels 1A–3A were judged as actionable mutations for molecular-targeted drugs^21^.

*p*-values ≤ 0.05 was considered statistically significant. All statistical analyses were performed using IBM SPSS Statistics (version 18.0; SPSS, Inc., Chicago, IL, USA).

### Ethics declarations

This retrospective study was approved by the Institutional Review Board of National Cancer Center Hospital (approval number 2017-136) and follows the ethical standards laid down in the Declaration of Helsinki. Informed consent was obtained from all patients.

## Results

**Figure 1:**
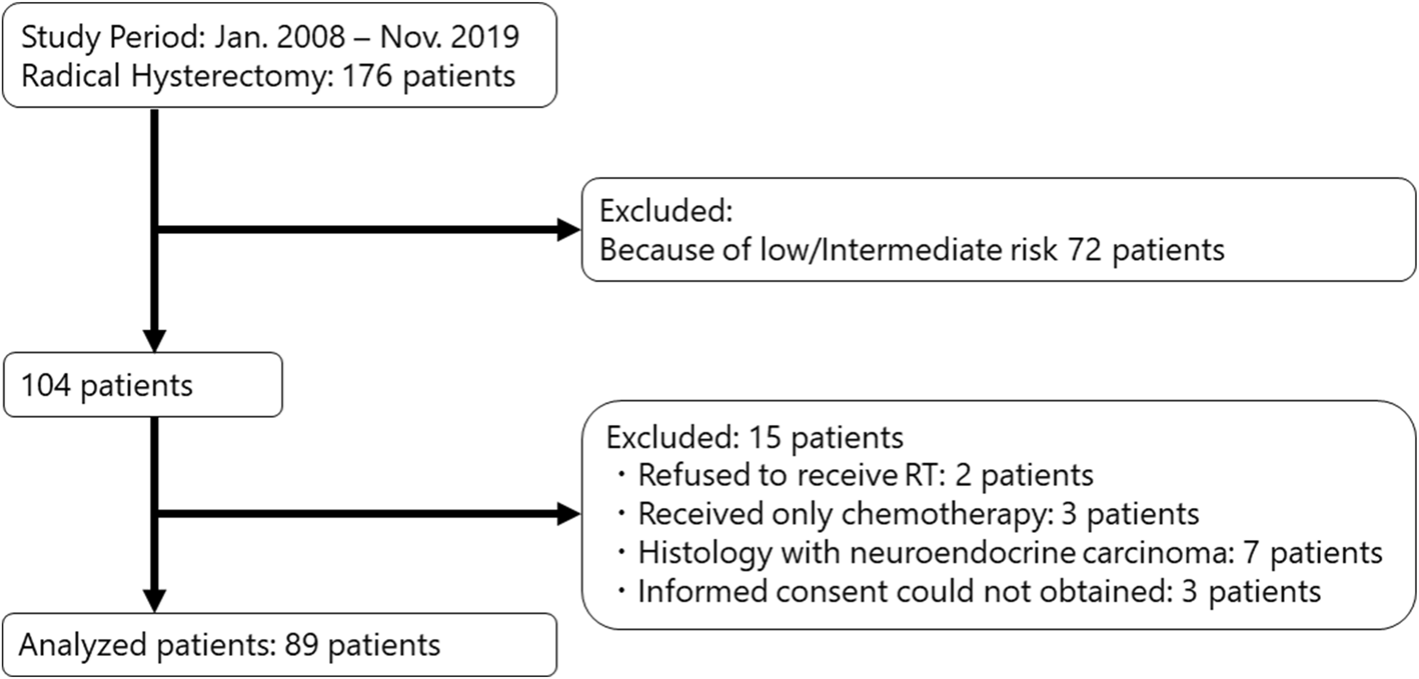
Patient flow diagram of this study.

**Figure 2:**
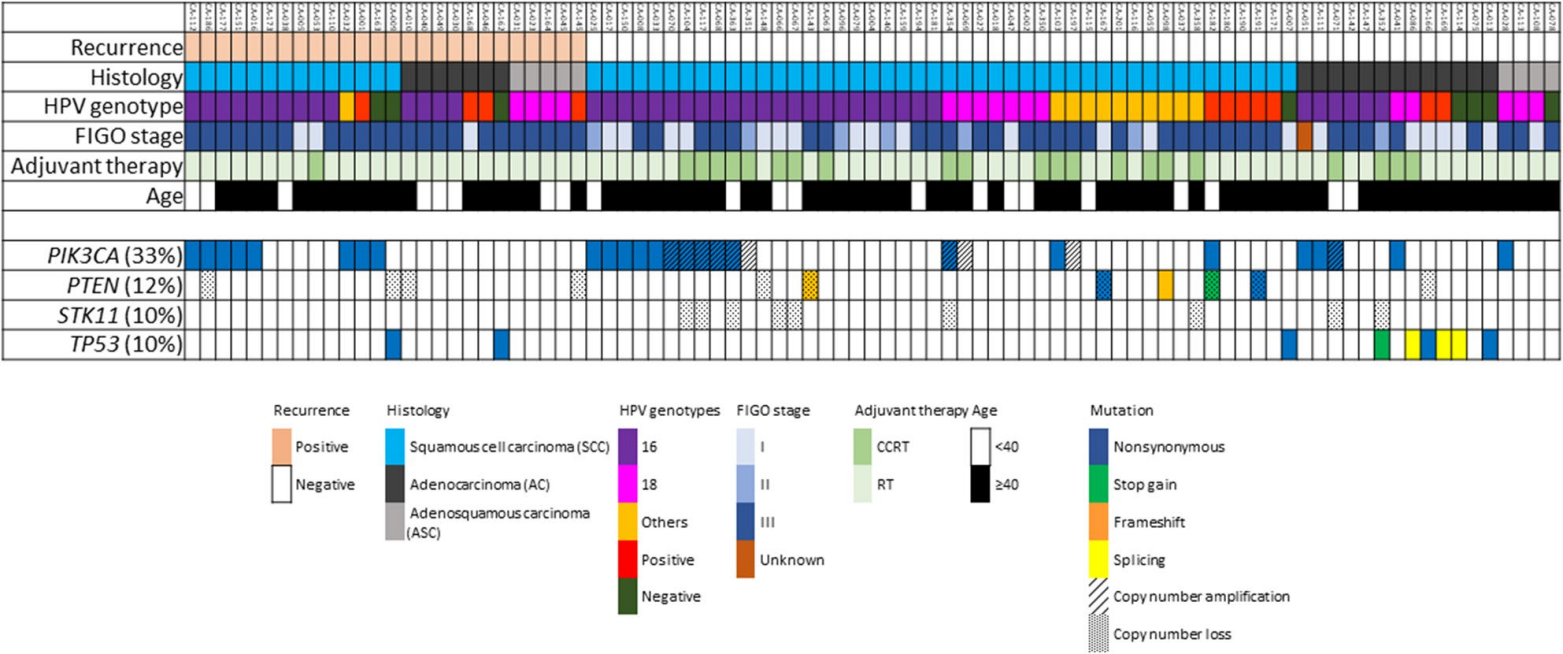
An overview of clinicopathological status and clinical outcomes of all patients included in this study. *Rec* recurrence.

The patient flow diagram of this study is shown in Fig. 1. From January 2008 to November 2019, 176 patients underwent abdominal radical hysterectomy for early-stage uterine cervical cancer, among which 92 patients underwent postoperative radiation therapy due to the presence of pathologically high-risk features. Informed consent could not be obtained from 3 patients; therefore, 89 patients were included in this analysis. Patient and tumor characteristics are summarized in Table 1, and an overview of the FIGO stage, pT classification, pN grade, pathology, and four most frequently mutated genes (*PIK3CA*, *STK11*, *PTEN,* and *TP53*) for each patient is summarized in Fig. 2. More than half of the patients had positive pelvic LN metastasis (64%) and parametrial involvement (66.3%). HPV infection was detected in 91.0% patients, of which 72.8% patients were infected with HPV-16 or HPV-18. Only 24.7% patients received CCRT as it was approved for use in 2015.

**Table 1:** Characteristics of 89 patients with cervical cancer.

Variable		n (%)
Age (year)	Median (range)	45 (26–70)
Histology	Squamous cell carcinoma	60 (67.4)
	Adenocarcinoma	20 (22.5)
	Adenosquamous carcinoma	9 (10.1)
FIGO stage	IA	0 (0%)
	IB	24 (27.0%)
	IIA	4 (4.5%)
	IIB	3 (1.1%)
	IIIA	0 (0%)
	IIIB	0 (0%)
	IIIC	57 (64.0%)
	Unknown	1 (1.1%)
Pathological T stage	Ia	0 (0%)
	Ib	15 (16.9%)
	2a	14 (15.7%)
	2b	60 (67.4%)
Lymph node metastasis	Positive	57 (64.0%)
	Negative	32 (36.0%)
Parametrial involvement	Positive	59 (66.3%)
	Negative	30 (33.7%)
Maximum tumor diameter (cm)	Median (range)	4.5 (1.7–8.7)
HPV	Positive	81 (91.0%)
	Negative	8 (9.0%)
HPV subtype	16 or 18	59 (72.8%)
	Others	22 (27.2%)
Treatment	RT alone	67 (75.3%)
	CCRT	22 (24.7%)
RT technique	3DCRT	26 (29.2%)
	IMRT	58 (65.2%)
	ICBT alone	5 (5.6%)

*RT* radiation therapy, *CCRT* concurrent chemoradiation therapy, *3D-CRT* three-dimensional radiation therapy, *IMRT* intensity modulated radiation therapy, *ICBT* intracavitary brachytherapy, *HPV* human papillomavirus.

The median follow-up period for living patients was 82.3 months (range 9.3–153.9). The 5-year relapse-free survival (RFS) and overall survival (OS) rates were 72.6% and 85.9%, respectively. Twenty-six patients developed tumor recurrence after primary abdominal radical hysterectomy followed by pelvic radiation therapy. Patterns of relapse are summarized in Table 2. In- and extra-field recurrence was observed in eight patients and 20 patients, respectively. Clinicopathological factors that had an influence on tumor recurrence or patient survival were analyzed using univariate and multivariate analyses (Table 3). In multivariate analysis, the presence of pelvic LN metastasis was identified as the independent prognostic factor for RFS (*p* = 0.005). Concurrent use of systemic chemotherapy showed trends toward better RFS in univariate analysis (*p* = 0.056). The presence of pelvic LN metastasis was associated with trends toward worse OS in univariate analysis (*p* = 0.08). The most frequent genetic alterations occurred in *PIK3CA* (29 [32.6%] patients) followed by *STK11* (9 [10.1%] patients) and *PTEN* (11 [12.4%] patients) (Table 4). We examined whether pelvic LN metastasis was associated with somatic genetic alterations or HPV infection status; however, there was no significant association between the presence of pelvic LN metastasis and genetic alterations or HPV infection (Table 4). We also examined whether the disease recurrence was associated with somatic genetic alterations or HPV infection status (Table 5). There was no significant association between disease recurrence and somatic genetic alterations or HPV infection status.

**Table 2:** Distribution of recurrence with regard to the radiation field.

Variable	n (%)
Any recurrence	26
**(1) In-field or extra-field**	
Both in-field and extra-field recurrence	2 (7.7)
In-field recurrence	6 (23.1)
Extra-field recurrence	18 (69.2)
**(2) Extra-field recurrence**	20
PALN	7 (35.0)
Lung	6 (30.0)
Others	7 (35.0)

*PALN* para-aortic lymph node.

**Table 3:** Hazard ratios for relapse-free survival and overall survival in cervical cancer.

Variable	Univariate		Multivariate	
	HR (95% CI)	*p-*value	HR (95% CI)	*p-*value
**Relapse-free survival**				
Age (≥ 45/ < 45)	0.94 (0.44–2.03)	0.88		
Histology (non-Scc/Scc)	1.86 (0.86–4.02)	0.12	1.84 (0.84–4.01)	0.13**
Size (≥ 4 cm/ < 4 cm)	1.38 (0.55–3.43)	0.49		
Parametrium involvement (positive/negative)	2.00 (0.76–5.32)	0.16	2.45 (0.92–6.56)	0.07**
Pelvic lymph node metastasis (positive/negative)	5.25 (1.57–17.5)	0.007*	5.64 (1.68–18.9)	0.005**
Concurrent use of systemic agent (CCRT/RT)	0.14 (0.02–1.05)	0.05	0.17 (0.02–1.25)	0.08**
*PIK3CA* alterations (positive/negative)	1.08 (0.47–2.52)	0.85		
*STK11* alterations (positive/negative)	1.77e−9 (–)	0.99		
*PTEN* alterations (positive/negative)	1.09 (0.37–3.21)	0.88		
HPV infection (positive/negative)	0.83 (0.25–2.77)	0.76		
HPV 16 or 18 (HPV 16/18/non-HPV 16/18)	1.58 (0.58–4.34)	0.37		
**Overall survival**				
Age (≥ 45/ < 45)	1.63 (0.48–5.59)	0.43		
Histology (non-Scc/Scc)	1.08 (0.32–3.69)	0.90		
Size (≥ 4 cm/ < 4 cm)	1.82 (0.39–8.44)	0.44		
Parametrium involvement (positive/negative)	1.22 (0.32–4.61)	0.77		
Pelvic lymph node metastasis (positive/negative)	6.51 (0.82–50.1)	0.08		
Concurrent use of systemic agent (CCRT/RT)	1.40e−9 (–)	0.99		
*PIK3CA* alterations (positive/negative)	1.35 (0.39–4.63)	0.63		
*STK11* alterations (positive/negative)	4.98e−9 (–)	0.99		
*PTEN* alterations (positive/negative)	0.73 (0.09–5.67)	0.76		
HPV infection (positive/negative)	1.24 (0.16–9.70)	0.84		
HPV 16 or 18 (HPV 16/18/non-HPV 16/18)	3.66 (0.46–28.9)	0.22		

*Scc* squamous cell carcinoma, *CCRT* concurrent chemoradiation therapy, *RT* radiation therapy, *HPV* human papillomavirus.

*Cox proportional hazard model.

**Adjusted for histology, pamametrium involvement, concurrent use of systemic agent, and pelvic lymph metasitasis.

**Table 4:** Association between lymph node metastasis status and frequency of genetic alterations or HPV status.

Genetic alterations/HPV status	Lymph node metastasis		*p-*value*
	Positive [n = 57, (%)]	Negative [n = 32, (%)]	
*PIK3CA* alteration (n = 29)	20 (35.1)	9 (28.1)	0.64
*PTEN* alteration (n = 11)	8 (14.0)	3 (9.4)	0.74
*STK11* alteration (n = 9)	5 (8.8)	4 (12.5)	0.72
HPV infection (n = 81)	52 (91.2)	29 (90.6)	1.00
HPV-16 or HPV-18 (n = 59)	36 (63.2)	23 (71.9)	0.44

*HPV* human papillomavirus.

*Fisher’s exact t-test.

**Table 5:** Association between recurrence and frequency of genetic alterations or HPV status.

Genetic alterations/HPV status	Recurrence [n = 26, (%)]	No recurrence [n = 63, (%)]	*p-*value*
*PIK3CA* alteration (n = 29)	8 (30.8)	21 (33.3)	1.00
*PTEN* alteration (n = 11)	4 (15.4)	7 (11.1)	0.72
*STK11* alteration (n = 9)	0 (0)	9 (14.3)	0.053
*TP53* mutation (n = 9)	2 (7.7)	7 (11.1)	1.00
HPV infection (n = 81)	23 (88.5)	58 (92.1)	0.69
HPV-16 or HPV-18 (n = 59)	18 (69.2)	41 (65.1)	0.59

*HPV* human papillomavirus.

*Fisher’s exact t-test.

**Figure 3:**
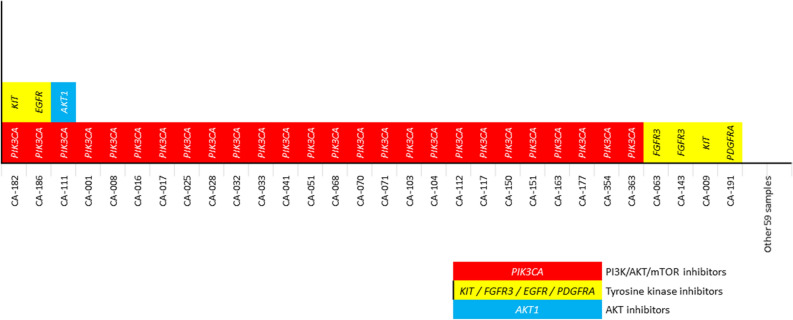
An overview of actionable genetic mutations in cervical cancers. CA-xx represents identification numbers for pathologic specimens of each patient.

The presence of actionable mutation was assessed based on the OncoKB evidence level; an evidence level of > 3A was regarded as having a potential target drug against the mutation. A total of 33 actionable mutations were detected in 30 (33.7%) patients. Among 29 patients with *PIK3CA* mutation, three patients had gene amplifications that were not regarded as actionable mutation according to the OncoKB evidence level; therefore, 26 patients with *PKC3CA* had actionable mutations in the current study. *PIK3CA* mutations accounted for most frequent actionable mutations (78.8%, Fig. 3).

## Discussion

In this retrospective study, the most frequent genetic alterations occurred in *PIK3CA* (32.6%) among patients with high-risk postoperative uterine cancer. The Phosphatidylinositol-3-kinase (PI3K)/AKT/mechanistic target of rapamycin (mTOR) pathway is a critical signaling cascade that plays several central regulatory roles such as cell growth, survival, and metabolism. PI3Ks are a family of lipid kinases comprising three classes that regulate signaling pathways involved in cell proliferation, cell differentiation, protein synthesis, glucose metabolism, transformation, cell migration, cell survival, apoptosis, and metastasis^22,23^. Molecular mutations in the PI3K pathway drive tumorigenesis in humans^24^. In this study, *PIK3CA* mutation was not associated with poor prognosis; however, it was a frequently altered pathway that can lead to cancer activation in > 70% cases of malignant tumors^25,26^. Moreover, activation of PI3K/AKT/mTOR signaling makes a tumor more aggressive in terms of proliferation, angiogenesis, metastasis, and drug resistance. *PIK3CA* mutations have also been reported in several malignancies such as breast cancer, head and neck cancer, glioblastoma, gastric cancer, lung cancer, prostate cancer, anal canal cancer, hepatocellular cancer, colorectal cancer, endometrial cancer, and cervical cancer^24,27^. The prognosis of breast cancer patients with *PIK3CA* mutations has been reported to be better than that of patients without *PIK3CA* mutations. In a recent phase III clinical trial, an oral *PI3K* inhibitor, alpelisib, in combination with fulvestrant prolonged the progression-free survival of patients with *PIK3CA*-mutated, hormone receptor-positive, human epidermal growth factor receptor 2-negative breast cancer^28^, suggesting *PIK3CA* as a candidate target for cervical cancer treatment. The mechanism of this particular treatment target needs to be elucidated in future studies.

In the RTOG9112/SWOG8797 trial, the 4-year OS rate of post-operative high-risk uterine cervical cancer patients in the CCRT arm was 81%^8^, while the 5-year OS rate in our study was 85.9%, which was comparable to the results of the RTOG9112/SWOG8797 trial despite the fact that only 24.7% patients received CCRT in our study. In our study, high-risk postoperative uterine cancer patients who received post-hysterectomy pelvic radiation therapy with concurrent weekly CDDP showed trends toward better RFS (hazard ratio, 0.14; 95% confidence interval, 0.02–1.05, *p* = 0.056), which was in line with the results of the RTOG9112/SWOG8797 trial, showing the superiority of RT with CCRT over RT alone.

As reported in previous studies^29–31^, the presence of pelvic LN metastasis was an independent adverse prognostic factor for RFS and no somatic mutation assessed by the cancer panel was associated with clinical outcomes in patients who received post-operative pelvic radiation therapy for uterine cervical cancer in the current study. In contrast, Yoshimoto et al. reported that *FGFR* mutation was associated with worse progression-free survival in cervical cancer patients treated with definitive radiation therapy^32^. The possible reason why *FGFR* mutation was not found to be a worse prognostic factor in the current study could be that the treatment modality was not the same between the two studies. In addition, the frequency of *FGFR* mutation was low, (< 5%) in this study. Although the prognosis of HPV-negative cervical cancer is worse than that of HPV-positive cancer^33^, HPV infection status did not affect prognosis in the current study (Table 3), possibly because the number of patients with HPV-negative cancer was not enough to detect differences.

In the current study, actionable mutations were observed in 33.7% patients. Currently, only bevacizumab, a molecular-targeted agent against vascular endothelial growth factor, is approved for recurrent or metastatic cervical cancer treatment^34^. Development of a targeted drug against *KRAS* mutation, considered a non-treatable mutation was believed to be difficult previously; however, an effective drug was finally developed^35^. Therefore, it is suggested that targeted drugs that are selected based on individual somatic mutations could be used in the treatment of uterine cervical cancer patients in the near future. Recently, the public insurance in Japan was updated, covering comprehensive somatic genetic alteration analysis for solid tumors. This means that the currently performed one-fits-all approach based on a specific organ will be replaced with personalized treatment strategies based on somatic genetic alterations for many types of solid cancers in the future. In this context, the current study provides important information for Asian uterine cervical cancer patients.

There are several limitations in this study. This study was a single-institution retrospective study with a limited number of patients. Further, due to the reduction of late gastrointestinal toxicities as result of improvement of radiation therapy delivery methods such as 3D-CRT to IMRT, the use of concurrent administration of weekly CDDP was approved in 2015. Therefore, the treatment performed in this study was inconsistent throughout the study period.

In conclusion, actionable mutations were detected in more than half of the patients, suggesting that precision medicine can be offered to post-operative high-risk uterine cervical cancer patients in the future.

## Data Availability

Although the authors performed next-generation sequencing, they are unable to upload these data, because they did not receive an agreement from all patients to register their sequence data in a database. However, the authors have described summary data of the somatic mutations in the paper. Interested researchers may send data requests to: Dr. Kouya Shiraishi (kshirais@ncc.go.jp).
